# Supplementary material for: Integrative and distinctive coding of visual and conceptual object features in the ventral visual stream
Source: eLife. 2018 Feb 2;7:e31873. doi: 10.7554/eLife.31873 (PMC5832413; doi:10.7554/eLife.31873)
Supplement: Transparent reporting form [file elife-31873-transrepform.docx]

***eLife’s* transparent reporting form**

We encourage authors to provide detailed information *within their submission* to facilitate the interpretation and replication of experiments. Authors can upload supporting documentation to indicate the use of appropriate reporting guidelines for health-related research (see [EQUATOR Network](http://www.equator-network.org/%20)), life science research (see the [BioSharing Information Resource](https://biosharing.org/)), or the [ARRIVE guidelines](http://www.plosbiology.org/article/info:doi/10.1371/journal.pbio.1000412) for reporting work involving animal research. Where applicable, authors should refer to any relevant reporting standards documents in this form.

If you have any questions, please consult our Journal Policies and/or contact us: [editorial@elifesciences.org](mailto:editorial@elifesciences.org).

**Sample-size estimation**

- You should state whether an appropriate sample size was computed when the study was being designed
- You should state the statistical method of sample size computation and any required assumptions
- If no explicit power analysis was used, you should describe how you decided what sample (replicate) size (number) to use

Please outline where this information can be found within the submission (e.g., sections or figure legends), or explain why this information doesn’t apply to your submission:

The behavior-based conceptual RDM and the behavior-based visual RDM were generated using data collected from 2846 participants. Of these, 1600 completed the conceptual feature generation task, 1185 completed the visual similarity rating task, and 61 (across tasks) were excluded for incomplete responding. These sample sizes are proportionally in line with those reported by McRae et al., (2005). This information can be found in the Methods section, second-level heading Participants, third-level heading Behavior-Based Visual Similarity Rating Task and Conceptual Feature Generation Task.

We did not complete a formal power analysis for the purpose of the fMRI study. However, selection of the targeted sample size (n = 16) was guided by extant fMRI studies that have used comparable analytical procedures to test hypotheses pertaining to object representation in the ventral visual stream, medial temporal lobe, and anterior temporal lobe (Bruffaerts et al., 2013 n = 19; Devereaux et al., 2013 n = 14; Martin et al., 2013, 2016 n = 18; Clarke and Tyler, 2014 n = 16; Erez et al., 2016 n = 19; Borghesani et al., 2016 n = 16; Chadwick et al., 2016 n = 18). This information can be found in the Methods section, second-level sub-heading Participants, third-level sub-heading Brain-Based fMRI Task.

**Replicates**

- You should report how often each experiment was performed
- You should include a definition of biological versus technical replication
- The data obtained should be provided and sufficient information should be provided to indicate the number of independent biological and/or technical replicates
- If you encountered any outliers, you should describe how these were handled
- Criteria for exclusion/inclusion of data should be clearly stated
- High-throughput sequence data should be uploaded before submission, with a private link for reviewers provided (these are available from both GEO and ArrayExpress)

Please outline where this information can be found within the submission (e.g., sections or figure legends), or explain why this information doesn’t apply to your submission:

Details regarding exclusion criteria used for behavior-based data are described in the Stimuli section.

We did not exclude any fMRI data.

**Statistical reporting**

- Statistical analysis methods should be described and justified
- Raw data should be presented in figures whenever informative to do so (typically when N per group is less than 10)
- For each experiment, you should identify the statistical tests used, exact values of N, definitions of center, methods of multiple test correction, and dispersion and precision measures (e.g., mean, median, SD, SEM, confidence intervals; and, for the major substantive results, a measure of effect size (e.g., Pearson's r, Cohen's d)
- Report exact p-values wherever possible alongside the summary statistics and 95% confidence intervals. These should be reported for all key questions and not only when the p-value is less than 0.05.

Please outline where this information can be found within the submission (e.g., sections or figure legends), or explain why this information doesn’t apply to your submission:

| **Behavioral Results** | | | | | | | | |
| --- | --- | --- | --- | --- | --- | --- | --- | --- |
| **Analysis** | **Location**  **in Text** | **N** | **Mean** | **SEM** | **Test** | **t-value** | ***P-*value** | **95% CI** |
| Comparison of response latencies between visual and conceptual feature verification tasks | Results – fMRI Task and Behavioral Results | 16 | Visual  1361 | Visual 75.79 | Paired  t-test | 1.002 | 0.333 | -49.90, 17.71 |
|  |  |  | Conceptual 1376 | Conceptual 79.00 |  |  |  |  |

| **Second-Level RSAs with Behavior- and Corpus-Based RDMs** | | | | | |
| --- | --- | --- | --- | --- | --- |
| **Analysis** | **Location**  **in Text** | **Kendall’s Tau-a** | **Statistical Test** | **SEM** | ***p* value** |
| Behavior-based visual RDM vs. behavior-based conceptual RDM | Results – Behavior-Based Similarity Models | 0.009216286494849 | Label Randomization Test  (10,000 permutations) | 0.006878556974986 | 0.097790220977902 |
| Behavior-based visual RDM vs. word2vec RDM | Results – Behavior-Based Similarity Models | 0.037253546624535 | Label Randomization Test  (10,000 permutations) | 0.013038110689016 | 8.999100089991385e-04 |
| Behavior-based conceptual RDM vs. word2vec RDM | Results – Behavior-Based Similarity Models | 0.110216911885718 | Label Randomization Test  (10,000 permutations) | 0.014082640584747 | 9.999000099991662e-05 |

| **Second-Level RSAs with Behavior- Corpus- and Brain-Based RDMs** | | | | | | | |
| --- | --- | --- | --- | --- | --- | --- | --- |
| **Comparison** | **ROI** | **Location** | **N** | **Kendall’s Tau-a** | **SEM** | **Wilcoxon’s Signed Rank** | **Exact *p* value** |
| Brain-based visual task RDM vs. behavior-based visual RDM | LOC | ROI-Based RSA: Comparison of Behavior-Based RDMs with Brain-Based RDMs | 16 | 0.045904813 | 0.011272963 | 120 | 0.002578735 |
| Brain-based visual task RDM vs. behavior-based conceptual RDM | LOC | ROI-Based RSA: Comparison of Behavior-Based RDMs with Brain-Based RDMs | 16 | -0.006022813 | 0.010425155 | 57 | 0.719055176 |
| Brain-based visual task RDM vs. word2vec RDM | LOC | ROI-Based RSA: Comparison of Corpus-Based RDMs with Brain-Based RDMs | 16 | 0.027504094 | 0.008475003 | 117 | 0.004592896 |
| Brain-based conceptual task RDM vs. behavior-based visual RDM | LOC | ROI-Based RSA: Comparison of Behavior-Based RDMs with Brain-Based RDMs | 16 | 0.006126731 | 0.007061055 | 90 | 0.13722229 |
| Brain-based conceptual task RDM vs. behavior-based conceptual RDM | LOC | ROI-Based RSA: Comparison of Behavior-Based RDMs with Brain-Based RDMs | 16 | 0.002897063 | 0.007065591 | 81 | 0.264083862 |
| Brain-based conceptual task RDM vs. word2vec RDM | LOC | ROI-Based RSA: Comparison of Corpus-Based RDMs with Brain-Based RDMs | 16 | -0.012124983 | 0.006428269 | 35 | 0.958374023 |
| Brain-based visual task RDM vs. behavior-based visual RDM | PHC | ROI-Based RSA: Comparison of Behavior-Based RDMs with Brain-Based RDMs | 16 | 0.004609438 | 0.007687522 | 81 | 0.264084 |
| Brain-based visual task RDM vs. behavior-based conceptual RDM | PHC | ROI-Based RSA: Comparison of Behavior-Based RDMs with Brain-Based RDMs | 16 | 0.009297069 | 0.012189081 | 81 | 0.264083862 |
| Brain-based visual task RDM vs. word2vec RDM | PHC | ROI-Based RSA: Comparison of Corpus-Based RDMs with Brain-Based RDMs | 16 | 0.010175039 | 0.004556374 | 104 | 0.032699585 |
| Brain-based conceptual task RDM vs. behavior-based visual RDM | PHC | ROI-Based RSA: Comparison of Behavior-Based RDMs with Brain-Based RDMs | 16 | -0.008117063 | 0.006998796 | 66 | 0.550033569 |
| Brain-based conceptual task RDM vs. behavior-based conceptual RDM | PHC | ROI-Based RSA: Comparison of Behavior-Based RDMs with Brain-Based RDMs | 16 | 0.045988919 | 0.011539001 | 121 | 0.002090454 |
| Brain-based conceptual task RDM vs. word2vec RDM | PHC | ROI-Based RSA: Comparison of Corpus-Based RDMs with Brain-Based RDMs | 16 | 0.05025747 | 0.011007607 | 126 | 0.000656128 |
| Brain-based visual task RDM vs. behavior-based visual RDM | PRC | ROI-Based RSA: Comparison of Behavior-Based RDMs with Brain-Based RDMs | 16 | 0.052094213 | 0.00832454 | 135 | 3.05E-05 |
| Brain-based visual task RDM vs. behavior-based conceptual RDM | PRC | ROI-Based RSA: Comparison of Behavior-Based RDMs with Brain-Based RDMs | 16 | 0.036194375 | 0.008416822 | 128 | 0.00038147 |
| Brain-based visual task RDM vs. word2vec RDM | PRC | ROI-Based RSA: Comparison of Corpus-Based RDMs with Brain-Based RDMs | 16 | 0.025157377 | 0.008847628 | 117 | 0.004592896 |
| Brain-based conceptual task RDM vs. behavior-based visual RDM | PRC | ROI-Based RSA: Comparison of Behavior-Based RDMs with Brain-Based RDMs | 16 | 0.034824313 | 0.010002552 | 121 | 0.002090454 |
| Brain-based conceptual task RDM vs. behavior-based conceptual RDM | PRC | ROI-Based RSA: Comparison of Behavior-Based RDMs with Brain-Based RDMs | 16 | 0.057271 | 0.010022999 | 135 | 3.05E-05 |
| Brain-based conceptual task RDM vs. word2vec RDM | PRC | ROI-Based RSA: Comparison of Corpus-Based RDMs with Brain-Based RDMs | 16 | 0.035208154 | 0.007607908 | 127 | 0.00050354 |
| Brain-based visual task RDM vs. behavior-based visual RDM | TP | ROI-Based RSA: Comparison of Behavior-Based RDMs with Brain-Based RDMs | 16 | 0.004650313 | 0.006729348 | 82 | 0.247711182 |
| Brain-based visual task RDM vs. behavior-based conceptual RDM | TP | ROI-Based RSA: Comparison of Behavior-Based RDMs with Brain-Based RDMs | 16 | 0.035348 | 0.007607642 | 129 | 0.000289917 |
| Brain-based visual task RDM vs. word2vec RDM | TP | ROI-Based RSA: Comparison of Corpus-Based RDMs with Brain-Based RDMs | 16 | 0.026925944 | 0.008061403 | 119 | 0.003143311 |
| Brain-based conceptual task RDM vs. behavior-based visual RDM | TP | ROI-Based RSA: Comparison of Behavior-Based RDMs with Brain-Based RDMs | 16 | 0.000581875 | 0.009329946 | 70 | 0.469940186 |
| Brain-based conceptual task RDM vs. behavior-based conceptual RDM | TP | ROI-Based RSA: Comparison of Behavior-Based RDMs with Brain-Based RDMs | 16 | 0.05077515 | 0.007330844 | 134 | 4.58E-05 |
| Brain-based conceptual task RDM vs. word2vec RDM | TP | ROI-Based RSA: Comparison of Corpus-Based RDMs with Brain-Based RDMs | 16 | 0.028992869 | 0.006904218 | 128 | 3.81E-04 |
| Brain-based visual task RDM. vs. brain-based conceptual task RDM | LOC | ROI-Based RSA: Comparisons of Brain-Based RDMs within ROIs | 16 | -0.007868750 | 0.004694922 | 41 | 0.920471191 |
| Brain-based visual task RDM. vs. brain-based conceptual task RDM | PHC | ROI-Based RSA: Comparisons of Brain-Based RDMs within ROIs | 16 | 0.008206000 | 0.005960906 | 91 | 0.126113892 |
| Brain-based visual task RDM. vs. brain-based conceptual task RDM | PRC | ROI-Based RSA: Comparisons of Brain-Based RDMs within ROIs | 16 | 0.062706438 | 0.008627981 | 135 | 0.000030518 |
| Brain-based visual task RDM. vs. brain-based conceptual task RDM | TP | ROI-Based RSA: Comparisons of Brain-Based RDMs within ROIs | 16 | 0.027784375 | 0.010917206 | 111 | 0.012481689 |
| Brain-based visual task RDM vs. brain-based visual task RDM | LOC-PHC | ROI-Based RSA: Comparisons of Brain-Based RDMs Across ROIs | 16 | 0.008637188 | 0.008603352 | 92 | 0.115600586 |
| Brain-based visual task RDM vs. brain-based visual task RDM | LOC-PRC | ROI-Based RSA: Comparisons of Brain-Based RDMs Across ROIs | 16 | 0.010076250 | 0.008459860 | 94 | 0.096405029 |
| Brain-based visual task RDM vs. brain-based visual task RDM | LOC-TP | ROI-Based RSA: Comparisons of Brain-Based RDMs Across ROIs | 16 | -0.007047563 | 0.013638554 | 53 | 0.783401489 |
| Brain-based visual task RDM vs. brain-based visual task RDM | PHC-PRC | ROI-Based RSA: Comparisons of Brain-Based RDMs Across ROIs | 16 | 0.006906563 | 0.009576217 | 96 | 0.079528809 |
| Brain-based visual task RDM vs. brain-based visual task RDM | PHC-TP | ROI-Based RSA: Comparisons of Brain-Based RDMs Across ROIs | 16 | 0.006390256 | 0.008843879 | 88 | 0.161254883 |
| Brain-based visual task RDM vs. brain-based visual task RDM | PRC-TP | ROI-Based RSA: Comparisons of Brain-Based RDMs Across ROIs | 16 | 0.009914313 | 0.009390745 | 98 | 0.064865112 |
| Brain-based conceptual task RDM vs. brain-based visual conceptual task RDM | LOC-PHC | ROI-Based RSA: Comparisons of Brain-Based RDMs Across ROIs | 16 | -0.007526800 | 0.007543481 | 50 | 0.825805664 |
| Brain-based conceptual task RDM vs. brain-based visual conceptual task RDM | LOC-PRC | ROI-Based RSA: Comparisons of Brain-Based RDMs Across ROIs | 16 | 0.003966188 | 0.011166958 | 80 | 0.280944824 |
| Brain-based conceptual task RDM vs. brain-based visual conceptual task RDM | LOC-TP | ROI-Based RSA: Comparisons of Brain-Based RDMs Across ROIs | 16 | 0.009124438 | 0.004604914 | 100 | 0.052291870 |
| Brain-based conceptual task RDM vs. brain-based visual conceptual task RDM | PHC-PRC | ROI-Based RSA: Comparisons of Brain-Based RDMs Across ROIs | 16 | 0.015648875 | 0.007074927 | 124 | 0.011611938 |
| Brain-based conceptual task RDM vs. brain-based visual conceptual task RDM | PHC-TP | ROI-Based RSA: Comparisons of Brain-Based RDMs Across ROIs | 16 | 0.009520063 | 0.007143431 | 113 | 0.044326782 |
| Brain-based conceptual task RDM vs. brain-based visual conceptual task RDM | PRC-TP | ROI-Based RSA: Comparisons of Brain-Based RDMs Across ROIs | 16 | 0.011167875 | 0.006211454 | 118 | 0.025268555 |

| **Within-object First-Level RSA** | | | | | | | |
| --- | --- | --- | --- | --- | --- | --- | --- |
| **Comparison** | **ROI** | **Location** | **N** | **Pearson’s *r*** | **SEM** | **Wilcoxon’s Signed Rank** | **Exact *p* value** |
| Within-object multi-voxel activity patterns across task contexts | LOC | ROI-Based RSA: Comparisons of Within-Object Multi-Voxel Activity Patterns Across Different Task Contexts | 16 | 0.007518767 | 0.005555592 | 85 | 0.201873779 |
| Within-object multi-voxel activity patterns across task contexts | PHC | ROI-Based RSA: Comparisons of Within-Object Multi-Voxel Activity Patterns Across Different Task Contexts | 16 | -0.007914123 | 0.006802284 | 46 | 0.873886108 |
| Within-object multi-voxel activity patterns across task contexts | PRC | ROI-Based RSA: Comparisons of Within-Object Multi-Voxel Activity Patterns Across Different Task Contexts | 16 | 0.041197935 | 0.009415467 | 124 | 0.001068115 |
| Within-object multi-voxel activity patterns across task contexts | TP | ROI-Based RSA: Comparisons of Within-Object Multi-Voxel Activity Patterns Across Different Task Contexts | 16 | 0.034092557 | 0.012127097 | 116 | 0.005493164 |

**Group allocation**

- Indicate how samples were allocated into experimental groups (in the case of clinical studies, please specify allocation to treatment method); if randomization was used, please also state if restricted randomization was applied
- Indicate if masking was used during group allocation, data collection and/or data analysis

Please outline where this information can be found within the submission (e.g., sections or figure legends), or explain why this information doesn’t apply to your submission:

This information does not apply to the current study.

**Additional data files (“source data”)**

- We encourage you to upload relevant additional data files, such as numerical data that are represented as a graph in a figure, or as a summary table
- Where provided, these should be in the most useful format, and they can be uploaded as “Source data” files linked to a main figure or table
- Include model definition files including the full list of parameters used
- Include code used for data analysis (e.g., R, MatLab)
- Avoid stating that data files are “available upon request”

Please indicate the figures or tables for which source data files have been provided:

Source data files have been provided for Figures 1, 2, 6, 7, and 8.
